# Spatial task context makes short-latency reaches prone to induced Roelofs illusion

**DOI:** 10.3389/fnhum.2014.00673

**Published:** 2014-08-29

**Authors:** Bahareh Taghizadeh, Alexander Gail

**Affiliations:** ^1^Sensorimotor Group, German Primate Center, Leibniz Institute for Primate ResearchGöttingen, Germany; ^2^Faculty of Biology and Psychology, Georg-August-UniversitätGöttingen, Germany; ^3^Bernstein Center for Computational NeuroscienceGöttingen, Germany

**Keywords:** reach movement, induced Roelofs effect, illusion, reference frame, allocentric, object-centered

## Abstract

The perceptual localization of an object is often more prone to illusions than an immediate visuomotor action towards that object. The induced Roelofs effect (IRE) probes the illusory influence of task-irrelevant visual contextual stimuli on the processing of task-relevant visuospatial instructions during movement preparation. In the IRE, the position of a task-irrelevant visual object induces a shift in the localization of a visual target when subjects indicate the position of the target by verbal response, key-presses or delayed pointing to the target (“perception” tasks), but not when immediately pointing or reaching towards it without instructed delay (“action” tasks). This discrepancy was taken as evidence for the dual-visual-stream or perception-action hypothesis, but was later explained by a phasic distortion of the egocentric spatial reference frame which is centered on subjective straight-ahead (SSA) and used for reach planning. Both explanations critically depend on delayed movements to explain the IRE for action tasks. Here we ask: first, if the IRE can be observed for short-latency reaches; second, if the IRE in fact depends on a distorted egocentric frame of reference. Human subjects were tested in new versions of the IRE task in which the reach goal had to be localized with respect to another object, i.e., in an allocentric reference frame. First, we found an IRE even for immediate reaches in our allocentric task, but not for an otherwise similar egocentric control task. Second, the IRE depended on the position of the task-irrelevant frame relative to the reference object, not relative to SSA. We conclude that the IRE for reaching does not mandatorily depend on prolonged response delays, nor does it depend on motor planning in an egocentric reference frame. Instead, allocentric encoding of a movement goal is sufficient to make immediate reaches susceptible to IRE, underlining the context dependence of visuomotor illusions.

## Introduction

Goal-directed, object-oriented reach movements require accurate localization of the target object, yet object localization can be prone to visual illusions. The fact that in many cases visual perceptual localization is more prone to illusions than immediate visuomotor responses (Smeets and Brenner, 2001) is typically taken as strong evidence for two functionally independent visual processing streams, a ventral “vision-for-perception” pathway, and a dorsal “vision-for-action” pathway (Goodale and Milner, 1992; see Schenk et al., 2011 and Westwood and Goodale, 2011 for recent reviews). Understanding the circumstances under which perceptual illusions do or do not affect motor performance can be highly informative about the nature of the two putative visual streams, and, more specifically, about the nature of visuospatial processing during sensorimotor transformations (Smeets et al., 2002). Here we re-investigate the induced Roelofs effect (IRE) in reach movements. In the IRE, the position of a task-irrelevant visual object induces a shift in localization of the target object. The IRE depends on the mode of the subjects’ behavioral response to indicate this position, e.g., key-presses vs. immediate reaches towards the target (see details below). This observation was originally taken as evidence for the dual-visual-stream or perception-action hypothesis (Bridgeman et al., 1997), attributing the IRE to ventral stream perceptual processing. A later, opposing view explained the IRE by a phasically distorted egocentric (object-to-self) reference frame—i.e., changes in space defined relative to the own body—attributing the IRE to dorsal stream processing along the vision-to-action pathway (Dassonville and Bala, 2004b). Here we expand on these findings by revisiting the IRE in a short-latency reach task. In the first experiment, different to previous studies, we varied the spatial task context in which reaches had to be performed. We distinguished reaches in an allocentric (object-to-object) reference frame, i.e., a task in which the reach goal location is defined relative to another object, from otherwise identical reaches in an egocentric reference frame, i.e., reach goals relative to the own body. We thereby test if the IRE can also be induced for immediate reaches to the target (typically considered an “action” task) if the spatial context of the task is modified. In a second experiment, we test if the IRE critically depends on a phasic distortion of an egocentric frame of reference or if it can also be induced by allocentric encoding.

The IRE probes the illusory influence of task-irrelevant visual context stimuli on the processing of task-relevant visuospatial instructions during movement preparation. Note that task-relevance here refers to whether a stimulus was instructive for subjects, independent of its effect on behavior. In a series of studies Bridgeman et al. (1997, 2000) showed that the position of a task-irrelevant visual object (frame) can induce a systematic shift in localization of visual targets. When the frame was laterally off-center relative to subjects’ mid-sagittal plane, i.e., the frame was shifted to the left or right with respect to the subjects’ body midline, subjects misjudged the position of targets presented inside the rectangular frame (Bridgeman et al., 1997). The mislocalization was in the opposite direction of the frame shift, i.e., if the frame was left of the midline then targets were mislocalized to the right, and vice versa. Target mislocalization occurred in two conditions. First, when subjects had to indicate target position by pressing response keys assigned to different targets. The keyboard was placed on the table in front of the subjects, and hence the keys were spatially incongruent to the actual target positions. Second, when subjects pointed to the memorized position of the target after an instructed delay (Bridgeman et al., 1997). Importantly, when subjects in the same task indicated the target position without instructed delay by either pointing to it (Bridgeman et al., 1997) or by directly reaching to jab at the target (Bridgeman et al., 2000), no IRE was observed. This discrepancy was originally interpreted as an indication of separate visuospatial representations for direct sensorimotor processing (immediate reaching or pointing without instructed delay) in the dorsal visual stream, compared to spatial cognitive or perceptual processing (verbal response, using response keys, or pointing with instructed delay) in the ventral visual stream. This dual-visual-stream or perception-action hypothesis of the IRE was based on two assumptions. First, only the perceptual “cognitive” ventral stream is prone to the IRE illusion. Second, only the immediate and directly target-aimed manual responses can be performed by direct egocentric sensorimotor processing in the dorsal stream. Symbolic responses (verbal response or pressing of response keys) and delayed memory-guided reaching and pointing, on the other hand, require ventral stream processing (Bridgeman et al., 1997, 2000). In case of visually instructed delayed reaching and pointing, the need for ventral stream processing arises from the assumption that the dorsal vision-to-action pathway is incapable of even medium-term (several seconds) memory storage of the required reach parameters, while immediate reaches can be processed by the dorsal stream alone, as further discussed below.

**Figure 1:**
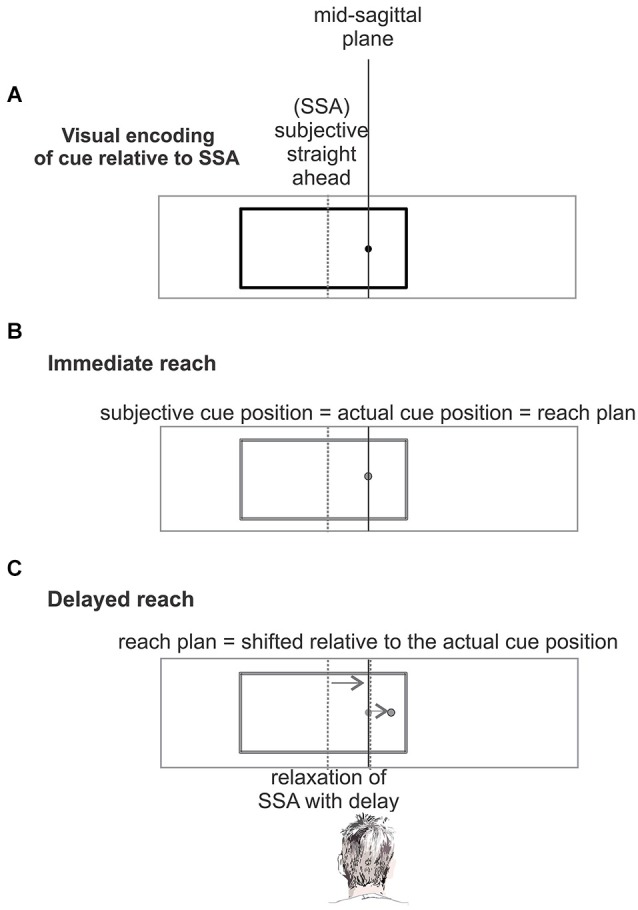
**The biased-midline hypothesis (Dassonville and Bala, 2004b). (A)** An off-center visual frame (black) shifts the subjective straight ahead (SSA, gray dashed line) in the direction of the frame. The location of a simultaneously presented cue is encoded in this distorted egocentric reference frame centered on the SSA. **(B)** In an immediate response task, after presentation of the cue and frame (panel **A**) the corresponding movement plan will be encoded and executed in the same shifted frame of reference and no mislocalization occurs. **(C)** In a delayed-response task, presentation of the cue and frame (panel **A**) is followed by a memory period. During the memory period, i.e., before movement execution, the temporarily biased SSA drifts back to the objective straight-ahead. The movement which was planned relative to the temporally biased egocentric reference will be executed relative to the original reference after relaxation of SSA back to objective straight-ahead and will show a localization error opposite to the direction of the frame offset.

Behavioral and imaging studies challenged this interpretation of the IRE in favor of an alternative biased-midline hypothesis (Dassonville and Bala, 2004b; Dassonville et al., 2004) in which the IRE is explained by a temporary distortion of the egocentric spatial frame of reference which is used for reach planning and which is centered on the direction of the subjective straight-ahead (SSA; see Figure 1). Dassonville and colleagues showed that the IRE can be accounted for by an observed mislocalization of the memorized array of reference positions, relative to which the target position had to be indicated with a saccade. Since the mislocalization of the memorized reference positions occurred in the same direction as the off-centered visual frame it explained the observed target localization error opposite to the off-centered frame. This finding was interpreted as indication of a phasic translational shift in an egocentric reference frame which is used for movement planning, and which is centered on the direction of SSA (Dassonville and Bala, 2004b; Dassonville et al., 2004). According to this biased-midline hypothesis, in an immediate motor response task (non-delayed pointing, reaching, or saccade) the target location and the corresponding movement plan will both be encoded in the phasically shifted egocentric frame of reference, and the movement plan will be executed while the reference frame is still shifted. No obvious movement error occurs, since movement planning and execution are both affected by the shift, and hence the shift is compensated (Figure 1B). In a delayed-response task, the movement will be executed after relaxation of the shifted SSA back to the mid-sagittal plane. This induces a target error to the direction opposite to the off-set visual frame, since the movement was planned relative to the SSA but executed relative to the original un-biased frame of reference after relaxation (Figure 1C).

An fMRI study of the IRE revealed differential activity in the dorsal visual stream but not in the ventral stream (Walter and Dassonville, 2008).The dual-visual-stream hypothesis would have pointed to a main contribution from the ventral stream for IRE-prone behavioral conditions. In contrast, the biased-midline hypothesis implies that the IRE is based on a single egocentric visuospatial reference frame, likely in the dorsal visual stream, which would be relevant for both the IRE-resistant “sensorimotor” or “action” tasks (immediate target-directed manual or ocular response) and the IRE-prone “cognitive” or “perceptual” versions of the task (delayed pointing and looking or symbolic responses). However, the localization of IRE-related neural activity in the dorsal stream does not answer the questions of which spatial reference frame and which temporal dynamics determine the behavioral consequences of the IRE. The previously suggested dual-visual-stream model for the IRE is tied to the perception-action model (Goodale and Westwood, 2004; Goodale et al., 2004), according to which the ventral and dorsal visual streams are preferentially associated with allocentric and egocentric processing, respectively. On the other hand, there is growing evidence for parallel existence of both spatial reference frames within the dorsal visual pathway (Burgess, 2006; Milner and Goodale, 2008) and it is clear that the brain uses both types of information for localization of spatial targets in many tasks (Byrne and Crawford, 2010). Accordingly, spatial locations are not purely encoded in egocentric frames of reference in the posterior parietal cortex. The fMRI-active areas in the Dassonville IRE study (Walter and Dassonville, 2008) overlapped not only with areas shown in previous experiments to be involved in egocentric spatial localization (Vallar et al., 1999), but also with areas involved in allocentric localization relative to immediate visual objects (Galati et al., 2000; Thaler and Goodale, 2011a) or the enduring spatial features of a familiar environment (Galati et al., 2010). In addition, Fink et al. (2003) showed that egocentric and allocentric (object-centered) reference frames can interact in the human parieto-frontal network. Although there are not many studies directly comparing egocentric and allocentric reference frame in monkeys, there is evidence that neurons in parietal area 7a can encode the spatial location of objects in an eye-centered (i.e., egocentric) reference frame (Andersen et al., 1985) as well as relative to other task-relevant objects (Chafee et al., 2007; Crowe et al., 2008). Neurons in the same area are gain-modulated by the position of the subject’s body in the surrounding environment (i.e., in world-centered reference frame) (Snyder et al., 1998). The original dual-visual-stream hypothesis for the IRE argued that the dorsal stream, which dominates immediate egocentric “action” tasks, makes use of the ventral stream information only in case of memory-guided tasks. This explains the susceptibility of reaches to the IRE when they are substantially delayed by several seconds (Bridgeman et al., 1997, 2000; Dassonville and Bala, 2004b).

In summary, both existing interpretations of the IRE, namely the dual-visual-stream and the biased-midline hypothesis, critically depend on the following observation: in tasks in which subjects are required to directly point to, look at, or touch the perceived target position, and in which they can do so in an egocentric reference frame, the IRE can be observed if the manual or ocular response is purposefully delayed by several seconds, but not if an immediate response is required (Bridgeman et al., 1997, 2000; Dassonville and Bala, 2004b). Since the biased-midline hypothesis assumes a distortion of an egocentric reference frame (a shifted SSA) which is only phasic, it predicts that immediate reaches should be resistant to IRE because visual encoding of the reference positions and the reach target are affected in the same way. The dual-visual-stream hypothesis, on the other hand, assumes that dorsal stream processing utilizes ventral stream information only in memory-guided action, hence, it predicts resistance to the IRE for immediate reaches in an egocentric reference frame, but makes no prediction about immediate target-aiming reaches in other reference frames. In experiment I we test if immediate reaches, independent of a prolonged reach delay, can become prone to IRE if the task context prevents egocentric reach planning. To dissociate egocentric from allocentric reach planning, we introduced a spatially incongruent object-centered reach task. In contrast to previous IRE reaching experiments, we also introduced ocular fixation constraints. Furthermore, the fact that the dorsal stream areas which are active during target localization in IRE tasks cover areas of egocentric as well as allocentric spatial encoding brings up the second and related question of whether the IRE is really restricted to phasic distortion effects on egocentric frames of reference induced by the relative position of an object to the body. If not, mislocalization effects like the IRE might also be induced by the relative (allocentric) position of an object relative to another object. Previous IRE experiments including allocentric task constraints were nevertheless still explained by egocentric causes (Dassonville and Bala, 2004b; Lester and Dassonville, 2011). In Experiment II we tested whether the IRE can interfere with allocentric reach planning and can thus be explained independently of an egocentric reference frame distortion.

## Materials and methods

### Apparatus

Subjects were seated in a dimly lit room in front of a fronto-parallel touch screen (43 cm distance from eye, screen center at eye level) so that their mid-sagittal plane was aligned to the center of the screen. Visual stimuli were presented on an LCD screen (19” ViewSonic VX922) mounted behind a touch-sensitive screen (IntelliTouch, ELO Systems, CA, USA). Custom-written display software (C++) was controlled via a real-time program running on a PXI computer (LabView, National Instruments). Stimulus display was synchronized with vertical synchronization of the screen to avoid latency jitter. Visual display latencies were recorded with a photo diode and corrected for during data analysis. All visual stimuli had a low intensity gray tone (9.0 cd/m^2^ on a 1.2 cd/m^2^ background) to minimize retinal afterimages. Hand position was registered using the touch screen. Gaze positions were registered using an infrared eye tracker at 500 Hz (SMI, Teltow, Germany, in experiment I and EyeLink 1000, Kanata, Canada, in experiment II). Subjects rested their head on a chinrest for stability. Behavioral parameters were monitored using the real-time control software.

### Subjects

All subjects had normal or corrected-to-normal vision and were naïve with regard to the purpose of the study. Detailed written instructions were given to the subjects before each experiment. Experiments were in accordance with institutional guidelines for experiments with humans and adhered to the principles of the Declaration of Helsinki. All subjects gave their informed written consent prior to their inclusion in the study.

Eleven right-handed subjects (20 to 27 years, four females) participated in experiment I and control experiment Ia. Nine different right-handed subjects (22 to 39 years, five female) participated in control experiment Ib. A disjunct group of subjects was necessary for this control task to avoid possible task interference with experiment I. Ten different right-handed subjects (16 to 27 years, five females) participated in experiment II and control experiment IIa.

### Experimental paradigm

The following procedures for implementing the IRE were common to both experiments. Details specific for the individual experiments, especially the spatial positioning of stimuli, will be described in experiments I and II below.

**Figure 2:**
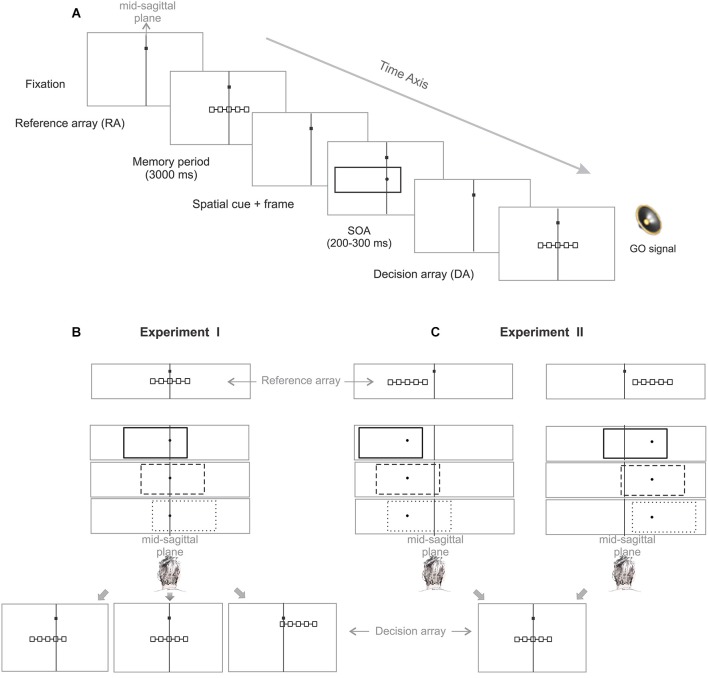
**Allocentric IRE task. (A)** Following successful eye and hand fixation, subjects are briefly presented a reference array (RA) consisting of five boxes indicating five potential positions for the upcoming cue. After a fixed memory period the cue (dot) was displayed simultaneously with a task-irrelevant context stimulus (frame). Subjects had to compare the position of the cue with the memorized reference positions indicated by the RA boxes to identify and reach to the corresponding target box within a decision array (DA) presented shortly afterwards. The DA was identical to the RA in size and shape but could appear at spatial locations congruent or incongruent to the RA. The vertical line within each frame indicates the subject’s mid-sagittal plane. **(B)** Experiment I: In order to test the IRE in an allocentric reference frame, we disentangled the position of the RA and DA for two-thirds of the trials. The congruency of the RA-DA was unpredictable to subjects in each trial. Therefore, to perform the task correctly, subjects had to encode the cue relative to the RA, i.e., use object-based (allocentric) spatial encoding. **(C)** Experiment II: In order to directly test the biased-midline hypothesis we disentangled the position of the RA from subject’s objective straight-ahead by randomly displaying the RA in either hemifield. The frame could take three different positions relative to the RA (allocentric shift of the frame to left, right or centered) for each RA location while it remained at the same side relative to the SSA (egocentric shift of the frame to the left/right for RA left/right location).

Each trial started with an eye-fixation target, presented to the subject at the vertical midline (mid-sagittal plane), and 5 cm (7° visual angle) above the horizontal midline of the screen (Figure 2A). Subjects were required to fixate the spot throughout each trial within an invisible window of 3 cm (4°) radius (ocular fixation). To start a trial, subjects had to push a “home” button, placed on subject’s mid-sagittal plane on the desk 40 cm below the screen center, and keep it pressed with their index finger until a “go” signal occurred later in the trial (manual fixation). Whenever subjects failed to keep ocular or manual fixation, the trial was aborted and repeated at a random later time during the experiment. After valid eye and hand fixation of 500–700 ms, a reference array (RA) of five boxes, horizontally connected with a line, appeared for 200 ms. Boxes were 0.35 cm (0.5°) squares, and centered 1.5 cm (2°) apart from each other. The position of the boxes indicated the potential positions of the pending cue. Subjects were required to keep these positions in mind for proper task performance, as will become clear below. Reference array presentation was followed by a memory period of 3 s. After the memory period a visual cue was presented for 200 ms. The cue consisted of a small dot of 0.27 cm (0.35°) diameter at the randomly chosen position of one of the five RA boxes, indicating the target box to which subjects should later reach. The cue was surrounded by a simultaneously presented frame, which was 16.9 cm (21°) wide and 6.6 cm (9°) high, but which was task-irrelevant. Cue and frame were succeeded by a decision array (DA), which was graphically identical to the RA, but was not necessarily spatially congruent (see below). Stimulus-onset asynchrony (SOA) between “cue + frame” and the subsequent DA was 200–300 ms. Simultaneously to the appearance of the DA, an acoustic signal was presented for 50 ms as the go-signal, indicating to the subject to lift their finger from the home button and touch the target position on the screen within 1000 ms after the go signal. After a correctly executed trial, subjects received acoustic feedback (high-pitched tone).

One constraint common to both experiments was that the frame could randomly take one of three possible positions relative to the RA: centered, or shifted by 3.85 cm (5°) to the left or right. Another constraint common to all experiments was that the cue appeared at one of the five RA positions. Subjects were instructed to hit the one of the five DA boxes which corresponded to the RA box at which they had perceived the cue, e.g., for a cue perceived at RA box #2 subjects should reach to DA box #2, irrespective of the absolute position of the DA. If the reach endpoint was within 4.5 cm (6°) distance from the target box the trial was counted as “successful”. By tolerating off-sets up to three boxes distant from the physically cued target box, we could analyze localization errors without inducing behavioral biases from error feedback. In the following sections, for each trial of the task the term “cue” refers to the dot stimulus presented simultaneously with the frame (Figure 2A, spatial cue + frame) and “target” refers to the position of the relevant box of the DA (i.e., the box of the DA that corresponds to the cued box of the RA).

Before entering the experiment, all subjects completed a training session and were encouraged to ask any questions which were not answered by the written instructions. The aim of the behavioral training was to familiarize subjects with object-based (allocentric) reach planning. More details on the training task will be elaborated for each experiment separately in the following sections.

### Experiment I

The main conclusion of this study will result from Experiment II. But since Experiment II differs in multiple respects from previous implementations of IRE tasks, we first wanted to establish some basic findings in our type of experimental setting to make the data more comparable to previous experiments. In experiment I, we asked what determines the “immediacy” of the reaches which previously did not show an IRE. Is it only the time between the presentation of the cue that instructs the target and the reach onset which determines whether the IRE occurs or not, or can the spatial frame of reference in which the cue and target have to be encoded cause an IRE even when other spatial and temporal reach parameters are matched? Experiment I and control experiment Ib aim to distinguish between these two alternatives by introducing a task in which reaches can be conducted without instructed delay (“temporally immediate”) but might be associated with a spatially non-congruent target position (“spatially not immediate”, Experiment I), or only with congruent target position as in previous experiments (Experiment Ib). It is important to note that the positions of the frame stimulus relative to the body are still at the straight-ahead direction as in the original Roelofs experiment and in previous IRE experiments. To be able to later dissociate the frame position from any egocentric frame of reference, body-centered or eye-centered, we also tested for the effect of ocular fixation in our task (control experiment Ia), which previous reach experiments did not do. Note also, the term “temporally immediate” refers to the fact that the visual stimuli necessary to determine the reach target were available to subjects earlier than typical reach responses would occur. This means that there was no major response delay imposed by the sequence of stimulus events. Although spatial stimulus-response incongruencies and the need for allocentric spatial encoding are known to induce reach response delays in the order of a few 10 ms (Gail and Andersen, 2006; Westendorff et al., 2010; Thaler and Goodale, 2011b; Westendorff and Gail, 2011), such short additional latencies are about two orders of magnitude less than the instructed delays necessary to evoke an IRE in previous experiments (Bridgeman et al., 1997; Dassonville and Bala, 2004b; Dassonville et al., 2004; Walter and Dassonville, 2006, 2008; Bridgeman and Hoover, 2008).

#### Methods of experiment I

In experiment I subjects were required to reach-to-touch the target location. The important difference of our design compared to previous IRE studies was that the physical positions of cue and target were spatially congruent in only 1/3 of the trials (Figure 2B). In the other trials the reference and DA were (partially) incongruent in their position, but otherwise identical. In experiment I the RA position was constant across trials and always at the center of the screen. The DA randomly took one of three possible positions relative to the RA: identical (congruent condition), shifted by 1.5 cm (2°) to the left (partly congruent), or shifted by 2.12 cm (2.8°) to the right and 2.12 cm up (incongruent). Only in the congruent condition were cue (one of the RA boxes) and target (the corresponding DA box) physically identical, as in previous IRE experiments using egocentric reaching or pointing tasks (Bridgeman et al., 1997, 2000; de Grave et al., 2002; Dassonville and Bala, 2004a,b; Dassonville et al., 2004; Walter and Dassonville, 2006, 2008; Bridgeman and Hoover, 2008; Lester and Dassonville, 2011). This task design resulted in 45 possible combinations of cue (target), frame and DA positions (5 × 3 × 3), which were randomly presented. Since subjects could not predict whether a trial will be congruent or not, they always had to encode cue position with respect to the RA in order to be able to perform the task correctly. Subjects needed to perform 200 hit trials, resulting in at least four repetitions per condition. In case subjects’ errors might not be balanced across conditions, we decided against using “pseudo-random” trial orders to avoid changing probabilities of individual task conditions. Instead, we presented more than 4 × 45 trials to yield a minimum of four repetitions per conditions. Analysis of exactly four trials per condition instead of 4–5 trials per condition did not change the results.

Training was identical to the experimental task, except that the frame was not presented. Training was terminated after 20 hit trials.

#### Methods of control experiment Ia

In a control experiment Ia, we tested whether the presentation of the ocular fixation target has an impact on the IRE. Since previous studies on IRE purposefully tried to avoid any possibility of allocentric spatial coding, no ocular fixation stimulus was shown to subjects during the trial (Dassonville and Bala, 2004b). Hence, in our control experiment Ia, we omitted the ocular fixation stimulus and did not impose any constraints on eye movements. This control was run for all subjects of experiment I on a separate day.

#### Methods of control experiment Ib

In control experiment Ib, we reproduced the original IRE paradigm (Bridgeman et al., 1997) in order to establish that our setup and task layout allows us to reproduce previous findings of no IRE in immediate reaches. We used an independent group of subjects to avoid a possible transfer of response strategy between the two experimental designs. Each trial started with ocular and manual fixation. After valid fixation, cue and frame were simultaneously presented. Following the offset of cue and frame, an acoustic go signal indicated to the subjects to lift their finger from the starting home button and reach-to-touch the perceived location of the cue. Subjects had 1000 ms to finish the reach and they were required to hold ocular fixation until the end of the trial. There were no reference or decision objects shown in control experiment Ib. Importantly, the spatial layout and timing of the stimuli was otherwise identical to experiment I, i.e., the same cue, target and frame positions, sizes and presentation times were used. The 15 different possible combination of cue and frame (5 cue locations (0, ±2° and ±4° relative to the mid-sagittal plane) and 3 frame locations (0 and ±5° relative to the mid-sagittal plane)) were randomly presented to the subjects. For six out of the nine subjects stimuli had 23.5 cd/m^2^ luminance on a 1.2 cd/m^2^ background, for the other three the contrast was identical to experiment I. The results were independent of stimulus contrast, hence will be presented jointly.

### Experiment II

In experiment II, we tested whether the IRE in experiment I can be explained by a biased perception of the SSA. After we established with experiment I that incongruent reference and DA positions encourage allocentric reach planning and allow an IRE for short latency reaches to the target, we now additionally dissociated the position of the RA from the straight-ahead direction to test explicitly whether the IRE is determined by frame position relative to straight-ahead or relative to the RA.

During the training session for experiment II subjects performed the identical task to the incongruent condition of experiment I, but without the frame stimulus. The goal was to familiarize subjects with the setup and the allocentric reach task. Training was terminated after 20 hit trials.

#### Methods of control experiment IIa

Trials in experiment IIa were identical to the incongruent condition of experiment I. Subjects conducted 75 correctly performed trials to test whether they were prone to IRE in the allocentric reach task. This served as baseline for the expected effect size in the experiment II for this group of subjects.

#### Methods of experiment II

In Experiment II, we dissociated the position of the RA from the objective straight-ahead (see Figure 2C). Except for the positions of decision and RA, the procedure was the same as in the experiment I. The RA was randomly shifted by 5.8° (4.5 cm) either to the left or to the right of the objective straight-ahead with equal probability. As an example, consider the case when the RA was shifted to the right by 5.8°. Even if the frame was shifted by the maximum value of 5° to the left relative to the center of the RA (leftward allocentric shift of the frame), the center-of-mass of the frame still remained in the same hemi-field relative to objective straight-ahead (rightward egocentric shift of the frame, see Figure 2C). Although the frame could take three different positions relative to each of the two RA positions, it always stayed to the right of the body’s midline if the RA was on the right side, and it stayed left of the body’s midline when the RA was on the left side. Subjects were asked to maintain ocular fixation on the fixation target at the objective straight-ahead direction to align the body-centered reference frame with the gaze-centered reference frame. The DA was always located at the center of the screen, i.e., at the objective straight-ahead direction in all trials. According to the biased midline hypothesis, an off-centered frame relative to the body midline will cause target mislocalization to the direction opposite the frame shift. Therefore, one would expect when the RA and the frame were placed in the left or right hemi-field, they would cause a shift of the SSA to the same direction as the egocentric shift of the frame, thereby causing mislocalization of cue or target to the opposite side (Figure 5A). The 30 possible combinations of target, frame and RA positions (5 × 3 × 2) were presented in random order. The experiment included 160 hit trials to achieve 4–5 repetitions per condition.

**Figure 3:**
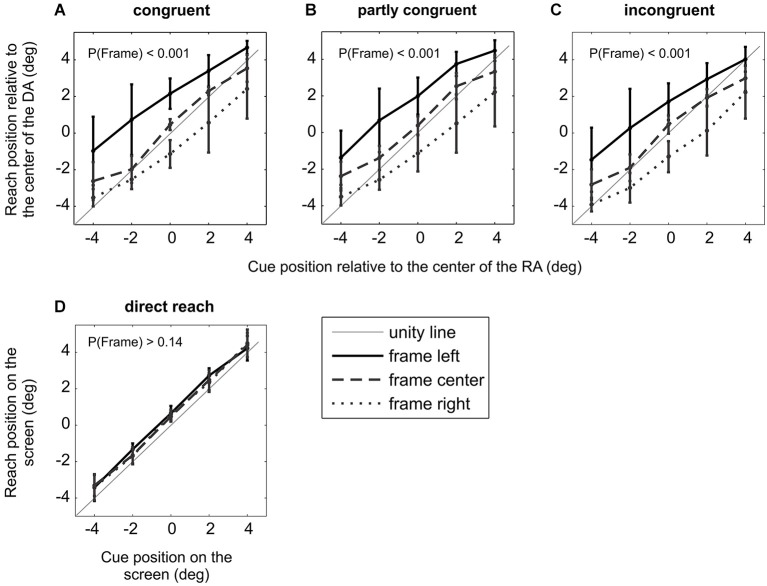
**Experiment I, Induced Roelofs effect (IRE) in immediate allocentric but not egocentric reach movements. (A)**–**(C)** Average effect of the frame off-set on the HRDA of 11 subjects. Data in the three panels show separately the three different congruency conditions between RA and decision array. Error bars represent S.E.M. For all three congruency conditions there was a significant main effect of the frame, indicating an IRE. **(D)** Replication of a previous finding (Bridgeman et al., 2000): there was no significant effect of the frame in immediate reach movements in which subjects were not required to use an object-based encoding for reach planning, i.e., when no task-relevant RA existed.

**Figure 4:**
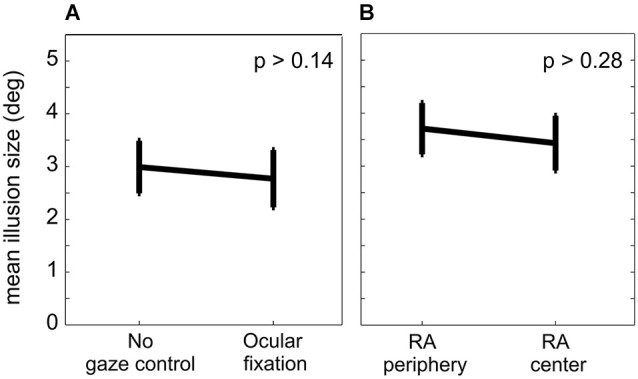
**Localization error in different conditions. (A)** Mean localization error across 11 subjects and three different congruency conditions with (experiment I) and without (control experiment Ia) ocular fixation. There was no significant difference between the mislocalization error between the two fixation conditions. **(B)** Mean localization error across 10 subjects for two lateral positions of RA. There was no significant difference in the mislocalization error between the RA in the periphery (experiment II) and in the center (control experiment IIa). Error bars represent S.E.M.

**Figure 5:**
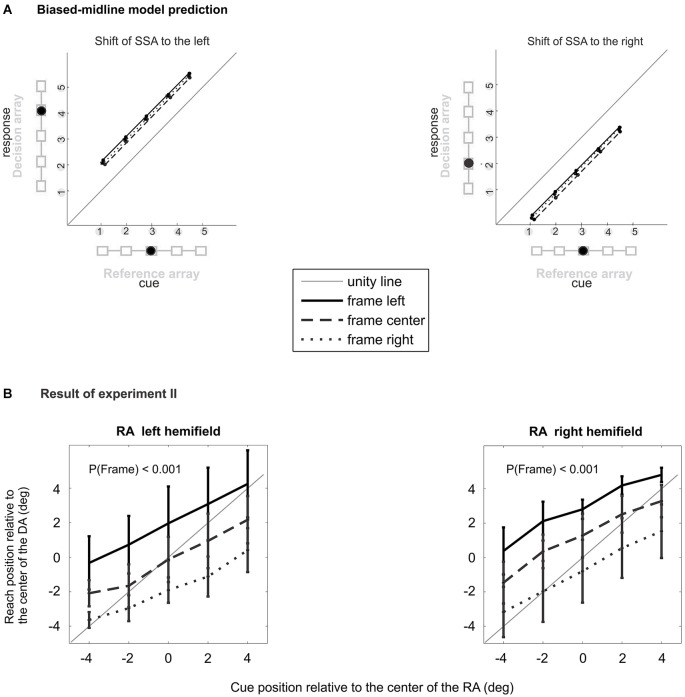
**Experiment II behavioral result. (A)** According to the biased-midline hypothesis the spatial layout of experiment II would cause mislocalization to the right/left for RA presented in left/right hemifield, respectively. **(B)** Effect of frame location on the relative average reach endpoint (HRDA) of 10 subjects separately for two positions of the RA indicates that an allocentric shift of the frame (shift relative to the RA) explains the mislocalization best. There was a significant main effect of frame and target location and no significant (frame × target) interaction.

### Data analysis

For each combination of target, frame and DA position, the horizontal reach endpoint relative to the center of the decision array (HRDA) was taken as the subject’s response (averaged across 4–5 identical trials). A HRDA of 2° (1.5 cm) means that the subject in this condition on average reached 1.5 cm to the right of the center of the DA. If the central box was cued, a HRDA of 2° (1.5 cm) corresponds to the nearest right neighboring box. A two-factor analysis of variance with cue position (5 levels) and frame position (3 levels) as factors was applied to HRDA for the population of all subjects (repeated measures ANOVA). A significant main effect of the factor “frame” indicated IRE. Additionally, for each position of the DA, the HRDA in the frame-right conditions was subtracted from the frame-left conditions for each target position and the mean difference was computed. This average localization error was used to compare effect sizes between different task conditions.

## Results

### Results of experiment I

Figures 3A–C shows the average target localization error, quantified by the mean HRDA (see Section Methods), across all 11 subjects. The three panels show separately the three different DA positions. All three DA conditions showed highly significant main effects of the factors “cue” (incongruent/partly-congruent/congruent: *F*_cue_(4,40) = 134/124/142, all *p*_cue_ < 0.0001) and “frame” (incongruent/partly-congruent/congruent: *F*_frame_(2,20) = 22.6/26.5/26.7, all *p*_frame_ < 0.0001), qualified by significant interactions (incongruent/partly-congruent/congruent: *F*_cue × frame_(8,80) = 5.80/6.02/5.55, all *p*_cue × frame_ < 0.0001).

The significant factor “frame” in all three DA conditions demonstrates that the IRE occurred independently of the trial-by-trial level of congruency between the reference and DA. The average localization error for individual subjects shows that the IRE was characterized by varying effect strength with most but not all subjects showing an IRE at the single subject level (average localization error for individual subjects: 3.79°, 4.38°, 0.52°,1.19°, 5.02°, 0.91°, 4.20°, 2.03°, 4.65°, 0.29°, 3.50°). The congruency condition did not affect the size of the localization error (*p* > 0.10, *F*_congruency_ = 2.57, two-factor repeated measure ANOVA on localization error for population of 11 subjects with factors “congruency” and “target relative to DA”). At the population level, the localization error was 2.77° (S.E.M. across subjects: 0.54°, S.E.M across all subjects and task conditions: 0.15°; Figure 4A).

This means that even in the congruent condition, which was identical to previous experiments in terms of spatial congruency of cue and reach target, a significant IRE was induced for immediate reaches. This was not the case in previous studies (Bridgeman et al., 1997, 2000; Dassonville and Bala, 2004b; Dassonville et al., 2004; Lester and Dassonville, 2011) where only congruent trials were presented (see also Section Results of control experiment Ib). None of the subjects showed a significant effect of congruency condition on reaction times (0.20 < *p* < 0.97, one-way ANOVA on per-subject trial-by-trial reaction times with factor “congruency”). From experiment I we can conclude that object-centered allocentric planning of immediate reaches is subject to the IRE.

In control experiment Ia we tested the effect of ocular fixation on the IRE by releasing the eye movement constraints but otherwise keeping everything identical to experiment I. The main result of this control was the same as for experiment I. The three congruency conditions in experiment Ia showed significant main effects of factors “cue” (incongruent/partly-congruent/congruent: *F*_cue_(4,40) = 98.4/97.0/99.7, all *p*_cue_ < 0.0001) and “frame” (incongruent/partly-congruent/congruent: *F*_frame_(2,20) = 38.2/32.2/34.4, all *p*_frame_ < 0.0001), qualified by significant interactions (incongruent/partly-congruent/congruent: *F*_cue × frame_(8,80) = 6.53/5.94/3.10, *p*_cue × frame_ <0.0001/<0.0001/<0.005). Mean localization errors for individual subjects were 5.45°, 6.22°, 0.40°, 2.77°, 6.24°,1.48°, 5.51°, 2.57°, 5.80°, 1.70°and 5.52°. Across the population of subjects, the localization error was 2.99° (S.E.M. 0.50°, Figure 4A). A two-tailed paired *t*-test between experiments I and Ia did not show a significant difference in localization error with and without ocular fixation (*p* > 0.14). From experiment Ia we can thus conclude that in our allocentric reach task the introduction of an ocular fixation constraint to align body- and gaze-centered reference frames does not affect the IRE.

In control experiment Ib we replicated the original finding of Bridgeman et al. (2000) for immediate reaches by asking subjects to reach and touch the perceived location of spatial cues which were presented within a frame (Figure 3D). The two-factor repeated measure ANOVA on the population of nine subjects showed a significant main effect of the factor “cue” (*F*_cue_(4,32) = 435, *p*_cue_ < 0.0001), but no significant effect of “frame” (*F*_frame_(2,16) = 2.15, *p*_frame_ > 0.14), qualified by a significant interaction (*F*_cue × frame_(8,64) = 2.27, *p*_cue × frame_ < 0.04). This means that the subjects correctly directed their reaches to the cue position (main effect of cue), but were unaffected by the frame stimulus (no main effect of frame). Correspondingly, mean localization errors for individual subjects were close to zero: 0.23°, −0.04°, 0.17°, 0.56°, 0.33°, 0.12°, −0.06°, −0.19°, 0.07°. The lack of an IRE for immediate egocentric reaches is comparable with the original finding (Bridgeman et al., 2000).

### Results of experiment II

In experiment I the sustained presence of a visual landmark at the direction of the objective straight-ahead, namely the fixation spot on which subjects kept ocular fixation, did not diminish the IRE. We consider it unlikely that despite continued ocular fixation at the true straight-ahead direction subjects would undergo a substantial shift in SSA. This allowed us to question the previous hypothesis that IRE is due to a temporarily perturbed perception of the SSA direction, an assumption of the biased-midline hypothesis that we want to test in Experiment II.

For both left and right peripheral positions of the RA, experiment II showed a significant effect of the factors “cue” (left/right: *F*_cue_(4,36) = 111/87.3, all *p*_cue_ < 0.0001) and “frame” (left/right: *F*_frame_(2,18) = 54.0/58.4, all *p*_frame_ < 0.0001), with no significant interaction (left/right: *F*_cue × frame_(8,72) = 1.63/1.69, *p*_cue × frame_ >0.13/ >0.11; Figure 5B). Individual subjects had mean localization errors of 5.08°, 4.20°, 2.54°, 6.09°, 5.75°, 5.91°, 6.25°, 6.73° and 0.34° in experiment II and 5.47°, 4.43°, 0.57°, 5.66°, 6.52°, 5.75°, 5.79°, 4.46° and 0.73° in control experiment IIa. The average localization error across subjects for peripheral RA in experiment II was 3.71° (S.E.M. 0.48°), and 3.43° (S.E.M. 0.52°) for the central RA in control experiment IIa (Figure 4B). A paired two-tailed *t*-test between test and control experiment did not reveal a significant difference (*p* > 0.28).

The result of experiment II shows that the main source of IRE in our data is the relative position of the frame with respect to the reference object (allocentric shift of the frame) rather than with respect to the straight-ahead direction (egocentric shift of the frame).

## Discussion

We conducted two experiments to study the effect of visual contextual information on reach planning. Our two experiments were designed such that subjects were required to encode first the cue and then the reach target relative to the position of a reference object, i.e., in an allocentric reference frame. In this case, subjects reliably showed an IRE (i) even for short-latency reaches to the target; (ii) with and without ocular fixation; and (iii) with mislocalizations being dependent on the allocentric position of the context stimulus (frame) relative to the center of the reference object, not the egocentric position relative to the SSA. Our results are not consistent with a previously suggested biased-midline hypothesis (Dassonville and Bala, 2004b). Instead, we suggest that the IRE can be induced by egocentric or allocentric spatial information, depending on which reference frame the task requires for the behavioral response.

### IRE for allocentric reach planning

In our study we show that IRE can be observed in an allocentric reference frame for reach planning, while previous studies emphasized the role of egocentric reference frames as an explanation.

Our findings argue against the idea that the IRE in our data can be explained by a phasic shift of the SSA direction (egocentric reference frame), as suggested previously (Dassonville and Bala, 2004b). First, we observed IRE with short-latency reaches. According to the biased-midline hypothesis short-latency reaches should not be subject to IRE since the assumed shift of the SSA is only phasic and affects target localization and reach planning likewise, such that the effect cancels out after relaxation of the SSA perturbation. Second, the fact that in our experiment I the IRE was also present when subjects were required to keep ocular fixation at a visual spot in the objective straight-ahead direction provided an additional hint that a shift in SSA might not be the cause of our observed results. We consider it rather unlikely that the SSA shifts in response to presentation of an off-center visual frame while subjects are fixating at a stable landmark in the true straight-ahead direction. Third, our experiment II provided direct evidence against the biased-midline hypothesis. For task conditions which should all have induced a SSA shift in the same direction, we found IRE in opposite directions (Figure 5). We therefore argue that in our data the Roelofs effect was not induced by an effect of the contextual visual frame stimulus on the SSA.

Ruling out a shifted SSA as explanation of the IRE in our experiment brings up the question which other egocentric or allocentric spatial encoding might be responsible for the observed IRE. Previous results do not contradict the idea of an allocentric IRE, since egocentric and allocentric reference frames were typically not dissociated. In previous IRE experiments (Bridgeman et al., 1997, 2000; de Grave et al., 2002; Dassonville and Bala, 2004a,b; Dassonville et al., 2004; Walter and Dassonville, 2006, 2008; Bridgeman and Hoover, 2008; Lester and Dassonville, 2011) subjects memorized the potential cue positions during a primary training period or behavioral calibration (i.e., equivalent to presentation of the RA in the present experiment). Later in the experiment or later in the trial subjects were asked to conduct reaches or saccades in which the egocentric encoding of the cue location was sufficient to solve the task. When subjects did not need to use the memorized positions to determine the target, no IRE was observed for immediate responses. But in such a task design, egocentric and allocentric references are aligned and the task-irrelevant visual frame is off-set equally in both reference frames. Therefore, even previous IRE task designs which required subjects to conduct a movement directly aiming at the target position, can in principle be consistent with an allocentric cause. Egocentric and allocentric representations of space are present in parallel and both types of information are usually used for more accurate behavior (Burgess, 2006; Byrne and Crawford, 2010). It has been shown that egocentric spatial memory is short lasting, putatively because it has to provide mainly real-time representation of the environment for direct interaction with objects (Hay and Redon, 2006; Chen et al., 2011). The fact that in previous task designs IRE was observed after a certain delay could be attributed to the interaction of short-lasting egocentric and long-lasting allocentric spatial representations. When the same subjects were exposed to a symbolic version of the task in which they had to use the memorized reference positions for a verbal response (to compare the position of visual cue with the memorized array of reference positions and report which one was cued), then the IRE was present even in immediate responses (Bridgeman et al., 1997; de Grave et al., 2002; Dassonville and Bala, 2004b). We argue that the verbal report of cue position required subjects to encode the cue relative to the RA hence mandated the use of an allocentric reference frame. It is therefore possible that even in previous IRE experiments the allocentric offset of the frame was the source of the illusion.

We suggest that the IRE in our reach task at least partially depended on allocentric encoding of space. Our present experimental design required subjects to follow an object-centered, hence allocentric, movement planning strategy. For proper interpretation of the IRE it is necessary to distinguish different phases of the trial when discussing reference frames. In the context of our task, at least the following spatial parameters are of interest: (i) the ego- or allocentric position of the (memorized) RA; (ii) the egocentric position of the frame relative to the body-midline; (iii) the allocentric position of the frame relative to the RA; (iv) the allocentric position of the cue relative to the memorized RA; and (v) the allocentric target position relative to the DA. Experiment II showed that the IRE was determined by the allocentric frame position relative to the RA, not the frame’s egocentric position. Thus, the IRE had an allocentric cause in our case. The consequence of this original allocentric cause needs to survive or be inherited by subsequent spatial encoding steps in order to affect the final motor behavior. The question is, which spatial encoding mediates the originally allocentric effect to finally become apparent in allocentric reach behavior? We ruled out a shifted SSA above. Previous studies showed that the memorized location of the reference object is shifted by the frame stimulus (Dassonville and Bala, 2004b). In our case this subjective shift of the RA would be sufficient to explain the results. The subjects need to encode the cue relative to the RA and later compute the target as the corresponding position on the re-located reference object (DA). Hence, a shifted RA translates into an erroneous allocentric cue position, and this in turn translates into an erroneous allocentric target position, and finally into an erroneous reach. Whether the memorized RA itself is encoded in an egocentric reference frame (e.g., relative to direction of gaze or body midline) or in an allocentric reference frame (e.g., relative to the surrounding screen frame) does not matter for the outcome. Both are possible and our experiment did not dissociate these alternatives.

### Expansion of memorized visual space

In all previous IRE studies, an underestimation of target eccentricity was reported along with a significant systematic mislocalization of the target. This can be seen by the fact that movement endpoint position as a function of cue position has a slope smaller than unity. The present results (Figures 3 and 5) also show underestimation of target eccentricity (*p*_experiment_ I < 0.0001 and *p*_experiment_ II < 0.0001, one tail *t*-test on the slope of the nine regression lines fitted separately to the population response for different DA and frame positions in experiments I and six regression lines fitted separately to the population response for different RA and frame positions in experiments II). In contrast to previous reports, subjects underestimated the object-centered eccentricity of the cue or target (i.e., laterality of the cue/target relative to the center of the reference/DA). Underestimated eccentricity can be viewed as an apparent compression of the movement space. Yet, when in a previous study subjects were asked to make saccadic eye movements to memorized reference locations, the apparent compression turned out to be a result of expansion of the spatial memory of potential target positions (Dassonville and Bala, 2004a). Our observed underestimation of eccentricity adds to previous findings by showing that expansion of memorized visual space occurs in the behaviorally relevant reference frame, here centered on the object.

### Perception vs. action

We do not argue that the behavioral report via an allocentric reach is necessarily substantially different from IRE tasks with symbolic encoding of the target, e.g., by key-presses or verbal report. The underlying mechanism of the IRE for this class of tasks, which previously were labeled “perceptual”, might be identical or at least overlap. Accordingly, previous lines of argumentation based on a perception-action model might also account for our data (see also Section Discussion on ventral and dorsal stream processing below). In this case, we would have to assume that the memorized RA underwent a “perceptual” shift due to the context stimulus (Dassonville and Bala, 2004b) with the consequences discussed in the above paragraph. Whether allocentric reach planning and perceptual encoding in the context of such tasks can at all be meaningfully distinguished, remains an open question. We find it noteworthy, though, that the congruent condition of the allocentric task (Experiment I, congruent trials) and the egocentric control condition (Experiment Ib and previous studies), were equivalent in terms of spatial layout, timing of stimuli, and manual response mode, and only differed in the task context requiring allocentric reach target selection. In terms of spatial layout, the equivalency refers to the fact that in congruent trials, the allocentric and egocentric spatial location of the cue (the dot which is presented with the frame) and the target (final reach goal) are identical. In terms of timing, equivalency refers to the fact that in both experiments subjects receive the acoustic go-signal soon after the presentation of the cue plus frame stimulus and faster than typical manual response times would require. In this sense the immediacy of the movement is given in both experiments. The task context was not provided by the congruent trials themselves but rather by the interspersed incongruent trials which requested subjects to encode the cue relative to the RA rather than according to their liking. If the congruent condition would have been predictable, the congruent trials could have been solved with egocentric encoding of the cue and target. This rendered the allocentric congruent trials, which showed an IRE, quite similar to the egocentric trials, which did not show an IRE. This means that spatial task context was enough to make short-latency reaches, which share many properties of typical “action” tasks, prone to IRE.

The results of experiment I differ from a recent study on IRE with an allocentric task in which the stimuli defining the allocentric reference frame were shown simultaneously with the off-center context stimulus, and no visual cue was shown to instruct the target (Dassonville and Bala, 2004b). The reach target was inferred in an allocentric reference frame as the fourth corner of a rectangle, while the other three corners were presented within a visual frame stimulus shifted laterally relative to the subject’s mid-sagittal plane. The pattern of observed target errors was similar to previous IRE experiments, with no effect for immediate responses and a significant effect for delayed responses. When the reference stimuli were shown together with an off-center frame, one had to expect that they will be subject to an IRE themselves (Lester and Dassonville, 2011) and the mislocalization of the target, which has to be inferred from the affected reference objects, is then a secondary effect without an IRE on the allocentric space representation itself. These results were used to argue against separate cognitive and sensorimotor visuospatial representations, and were instead explained with the biased-midline hypothesis, i.e., by an egocentric cause, an explanation that does not work for our data.

Taken together, we conclude that in our task, which required reach planning in an allocentric reference frame, the IRE was caused by an allocentric space representation and mediated via a distorted visual memory of the reference object. This may also have been the case in previous Roelofs experiments. It cannot be ruled out that an egocentric mislocalization of the memorized RA gave rise to the allocentric mislocalization of the visual cue, but it can be ruled out that the original cause for the mislocalization was a shift of the SSA direction or any other egocentric reference frame.

### Ventral vs. dorsal visual streams

According to the perception-action model (Goodale and Westwood, 2004; Goodale et al., 2004), egocentric references support visually guided actions through the dorsal sensorimotor stream in the posterior parietal cortex, while allocentric encoding of spatial locations can be predominantly found in the ventral stream supporting perception. According to this view, the dorsal stream is required and capable of making use of allocentric information from the ventral stream in the case of memory guided movements, e.g., IRE pointing tasks with long delays (Bridgeman et al., 1997; Goodale and Westwood, 2004; Milner and Goodale, 2008). In terms of the short-latency manual interaction with the visual target stimulus, our task would have to be considered a typical “action” task, hence should be attributed to dorsal stream processing. But according to the perception-action model, the allocentric spatial task constraints in our task also require ventral stream input. The model does not provide threshold values of how quickly ventral stream information can become accessible to dorsal stream processing. But in previous experiments, the required delays in target-aiming pointing, reaching, or saccade tasks ranged in the order of seconds before an IRE became apparent, suggesting a very slow transfer of information between ventral and dorsal stream in IRE tasks. If the model does account for our data, then our results suggest that the use of allocentric ventral-stream information by dorsal stream visuomotor processing can occur much faster than thought from previous IRE experiments. Such fast transfer is also suggested by a recent behavioral study (Thaler and Goodale, 2011b) which showed that reaction times in allocentric movements are 30–40 ms slower than egocentric movements, a finding that is reminiscent of behavioral and neural response delays in posterior parietal cortex during stimulus-response incongruent reach tasks (Gail and Andersen, 2006; Westendorff et al., 2010; Westendorff and Gail, 2011).

Slow brain imaging techniques cannot resolve the issue of whether such short-latency ventral-to-dorsal information transfer occurs, but experimental results have repeatedly pointed to overlapping structures for egocentric and allocentric encoding in the dorsal stream (Galati et al., 2000; Zaehle et al., 2007; Thaler and Goodale, 2011a; Gallivan et al., 2013). From our own previous neurophysiology work, we know that posterior parietal cortex encodes memory-guided anti-reach goals, which are independent of immediate visual input and independent of visual memory, with a delay of roughly 200 ms relative to visual cue onset, and roughly 100 ms relative to the visually selective neural response onset in the same area (Gail and Andersen, 2006; Gail et al., 2009; Westendorff et al., 2010). From the above discussion, we expect similar latencies for allocentric encoding in the posterior parietal cortex in the context of the current task.

The extent to which the perception-action model is valid is an ongoing debate in visual and visuomotor neuroscience. Growing evidence from behavioral and neurophysiology studies challenges the strictly separated vision-for-perception and vision-for-action theory (see Schenk et al., 2011 for review). The most compelling evidence for this model was patient D.F., who has bilateral damage to the ventral stream. D.F. failed in visual perceptual tasks while her visuomotor performance was not impaired (Milner et al., 1991). A recent study (Schenk, 2006) revealed that the discrepancy in her behavior was not due to different response modes, but rather due to deficits in different spatial representations (Himmelbach et al., 2012). The study showed that her perceptual performance was as good as her visuomotor performance when the perceptual task demanded egocentric spatial encoding whereas she failed in perceptual tasks which required object-based (allocentric) spatial encoding. Further behavioral support for the perception-action model was provided by a substantial body of research exploring visual illusions in perceptual and motor tasks where unlike perceptual responses, immediate motor responses seemed to be robust to the erroneous effects of spatial contextual information (for recent reviews see Schenk et al., 2011; Westwood and Goodale, 2011). However, in more controlled experimental conditions, contextual information can similarly affect perceptual and motor responses (Glover, 2004; Coello et al., 2007; Neely et al., 2008; Schenk et al., 2011). Therefore, based on our IRE for short-latency reaches, we suggest that the differential effect of spatial contextual information on sensorimotor behavior as explained by the perception-action model might not primarily be a question of perceptual vs. action-like behavioral response mode, but rather a question of the spatial task demands.

## Conflict of interest statement

The authors declare that the research was conducted in the absence of any commercial or financial relationships that could be construed as a potential conflict of interest.
