# Corrigendum: Spatial task context makes short-latency reaches prone to induced Roelofs illusion

**DOI:** 10.3389/fnhum.2014.00923

**Published:** 2014-11-14

**Authors:** Bahareh Taghizadeh, Alexander Gail

**Affiliations:** ^1^Sensorimotor Group, Cognitive Neuroscience Lab, German Primate CenterGoettingen, Germany; ^2^Faculty of Biology and Psychology, Georg-August-UniversitätGöttingen, Germany; ^3^Bernstein Center for Computational NeuroscienceGöttingen, Germany

**Keywords:** reach movement, induced Roelofs effect, Illusion, reference frame, allocentric, object-centered

The authors regret errors in the reported subject numbers and values for the illusion size in the data for individual subjects. This affects experiments Ia, Ib, II, and IIa. This mistake does not affect any of the conclusions of the paper since the reported mean values and standard errors were all correct. Correct values for the illusion size in the *N* individual subjects for each experiment are as follows:

E Ia (*N* = 11):
  4.13° 4.69° 0.30° 2.08° 4.70° 1.11° 4.15° 1.93° 4.37° 1.27° 4.16°
E Ib (*N* = 9):
  0.17° −0.03° 0.13° 0.42° 0.25° 0.09° −0.04° −0.14° 0.06°
E II (*N* = 10):
  3.83° 3.16° 1.91° 4.76° 4.59° 4.33° 4.45° 4.71° 5.07° 0.26°
E IIa (*N* = 10):
  4.12° 3.33° 0.43° 4.69° 4.26° 4.91° 4.33° 4.36° 3.36° 0.55°

## Conflict of interest statement

The authors declare that the research was conducted in the absence of any commercial or financial relationships that could be construed as a potential conflict of interest.

